# Experimental and Theoretical Tests on the Corrosion Protection of Mild Steel in Hydrochloric Acid Environment by the Use of Pyrazole Derivative

**DOI:** 10.3390/ma16020678

**Published:** 2023-01-10

**Authors:** Maria Boudalia, R. M. Fernández-Domene, L. Guo, S. Echihi, M. E. Belghiti, A. Zarrouk, A. Bellaouchou, A. Guenbour, J. García-Antón

**Affiliations:** 1 Laboratory of Materials, Nanotechnology and Environment, Faculty of Sciences, Mohammed V University in Rabat, P.O. Box 1014, Rabat 10090, Morocco; 2 Ingeniería Electroquímica y Corrosión (IEC), Instituto Universitario de Seguridad Industrial, Radiofísica y Medioambiental (ISIRYM), Universitat Politècnica de València, Camino de Vera, s/n, 46022 Valencia, Spain; 3 Departamento de Ingeniería Química, Universitat de València, Av. de les Universitats, s/n, 46100 Burjassot, Spain; 4 School of Material and Chemical Engineering, Tongren University, Tongren 554300, China; 5 Laboratory of Materials Engineering for the Environment and Natural Resources, Faculty of Sciences and Techniques, University Moulay Ismail of Meknes, BP 509 Boutalamine, Errachidia 52000, Morocco; 6 Laboratoire de Chimie Physique des Matériaux, Faculté des Sciences Ben M’Sick, Hassan II University of Casablanca, Casablanca 20000, Morocco; 7 Laboratory of Nernest Technology, 163 Willington Street, Sherbrooke, QC J1H5C7, Canada

**Keywords:** corrosion inhibitor, weight loss, electrochemistry, density functional theory, Monte Carlo simulation

## Abstract

In this study, 1,5-diallyl-1H-pyrazolo [3,4-d] pyrimidin-4 (5H)-one (PPD) was evaluated as an anticorrosion agent for mild steel (MS) in 1 M HCl. The analysis was performed by weight loss (WL), potentiodynamic polarization measurement, and electrochemical impedance spectroscopy (EIS). The Tafel polarization showed that PPD is a mixed-type inhibitor and reaches 94% of the protective efficiency at 10^−3^ M. EIS results indicated that the resistance to charge transfer increases with increasing inhibitor concentration and the corrosion of MS is controlled by a charge transfer process. The inhibitor adsorption on the MS surface obeyed the Langmuir’s adsorption isotherm. Thermodynamic parameters were calculated to elaborate the corrosion inhibition mechanism. The micrographic analysis revealed the existence of a barrier layer on the electrode surface with the presence of PPD. Theoretical examinations performed by electronic/atomic computer simulations confirmed that the obtained results were found to be consistent with experimental findings.

## 1. Introduction

Corrosion is considered to be more than an inevitable natural process. It is very crucial from an economic and environmental point of view, and the damage caused by rust has attracted remarkable attention owing to its impact on materials [1]. Hydrochloric acid solutions are commonly used, for example, in pickling, industrial acid cleaning, and oil well acidifying which are all processes that destroy metals. Mild steel corrodes severely during these processes, especially with the use of hydrochloric acid, which is a terrible waste of resources and money [2,3]. To minimize the effect of corrosion, there has been considerable industrial and scientific interest in studying corrosion control, in order to develop new, more economical and effective compounds that are often added to the acidic solution [4,5]. Valuable control methods have been used that can act through different mechanisms, thus offering better performance [6,7,8]. Many recent approaches have been used to protect MS from corrosion, such as sol-gel coatings of Glycidyloxypropyltrimethoxy alone and a sol-gel mixture of 3-glycidyloxypropyltrimethoxysilane-aminopropy trimethoxysilane (GPTMS-APTMS), which have been prepared and used to reduce wear on mild steel. The results showed that the silane coatings had excellent adhesion and corrosion resistance of over 1300 ohm/cm^2^ with an efficiency of 98% [9]. The performance of two different sizes of galvanic magnesium anodes on the cathodic protection of mild steel in 0.5 M hydrochloric acid was evaluated by electrochemical methods. The calculation of the corrosion rate was improved and subsequently the magnesium anodes were found to be effective as sacrificial anodes by up to 70% [10].

Recently, 50%BaO-*x*%V_2_O_5_-(50-*x*)%P_2_O_5_ (0 ≤ *x* ≤ 40 mol %) glass inhibitor revealed an inhibitive effect in 1 M HCl [11]. The inhibition efficiency depends on the V_2_O_5_ content in the glass system, which was assigned to a change of the nature of the phosphate groups in the glass with varying V_2_O_5_ content, where metaphosphate contributes the most to inhibition and exhibited superior inhibition efficiency of 90% for mild steel in acid medium. The *x*BiPO_4_-(90-*x*)P_2_O_5_-10NbOPO_4_ (10 ≤ *x* ≤ 35 mol %) system also showed an increase in protection capacity of up to 85% with increasing inhibitor concentration, which is of a mixed type [12]. This inhibitor tested was found to follow the Langmuir isotherm of adsorption and has affected both cathodic and anodic reactions. Further scientists have been interested in the introduction of rosin-based non-ionic surfactants as a corrosion inhibitor for steel pipes during acidizing of oil and gas wells. Inhibition efficiency increased and attained 92% [13].

Moreover, plant extracts, as natural inhibitors, have received greater scientific and industrial interest in the development of material wear control due to their cost effective and environmentally friendly aspects. The extract of *Artemisia herba alba* was explored for the inhibition of steel corrosion in phosphoric medium. The approach followed showed that the impedance of the inhibited substrate increases with the concentration of the inhibitor, and leads to a good inhibitory performance up to 88% at 1 g L^−1^, which may be the result of an increase in the surface coverage by the inhibitor molecules [4].

During the past few years, a variety of organic compounds have been reported as effective and economical methods of protecting the surface of the transition element Fe from dissolution. Several works have reported the effectiveness of heterocyclic compounds such as pyrazole, triazole, benzimidazole, pyrrole, and pyridine derivatives as corrosion inhibitors of metals in acidic solutions [14,15,16].

It is evident that heterocyclic compounds, containing electronegative functional groups, p-electrons and heteroatoms such as sulphur, nitrogen, and oxygen, as well as aromatic rings in their structures, often exhibit good corrosion inhibition capability in acidic media [14,17]. Electron-rich structures serve as potential adsorption sites, due to their ability to share free electrons with the metal substrate surface, and are considered to be responsible for the adsorption process of compounds that then provide greater efficiency, while also creating a barrier to the attack of the corrosive agent [15,16]. This can occur either by physical or chemical adsorption or sometimes by both ways, thus preventing the dissolution of the metal in an aggressive medium.

In recent decades, much research has been undertaken to further explain the effectiveness of these heterocyclic compounds, particularly the pyrazole compounds, in certain acidic destructive environments. However, pyrazole derivatives constitute an important class of heterocyclic compounds due to their applications in various fields.

Pyrazole derivatives are well known for their potential biologically active compounds; they have been synthesized and approved in medical chemistry as antimicrobial, pesticidal, antituberculosis, anti-inflammatory, etc., and are used as dyes, fungicides, herbicides, insecticides, and pesticides [18,19]. Due to these advantages, pyrazole compounds can be regarded as suitable candidates for corrosion inhibition of steel in acidic environments.

Several studies have illustrated the inhibition capacity of pyrimidine pyrazoles, in particular, on the corrosion of metals and alloys [20,21]. Recently, four pyrazole derivatives (PYRs) differing in the nature of their substituents were synthesized in an eco-friendly manner and their inhibition of acid corrosion was studied using experimental and computational approaches. The results proved the high inhibition efficiency of PYRs, which was dependent on the concentration and substituent [22]. A new heterocyclic pyrazole derivative was synthesized and used as a protective agent for steel. The study showed a high capacity to inhibit the corrosion reaction of steel as a mixed-type inhibitor at 400 ppm with a percentage of 90%. Theoretical quantum investigation revealed that this compound was an electron, while the steel surface was an electron acceptor [23]. Pyrazoles have also been reported as good corrosion inhibitors in a study of three pyrazolo-pyrimidine derivatives on copper in 0.5 M H_2_SO_4_. The results concluded that these pyrazoles effectively suppressed copper corrosion and their performance ranged from 70 to 96% [14]. The action of two pyrazole carboxamides referred to as DPC-1 and DPC-2. Both inhibitors were effective for steel alloys in a 1 M HCl solution. DPC-1 and DPC-2 had a maximum corrosion inhibition efficiency of 84.56% at 4 × 10^−4^ M and 80% at 1.6 × 10^−4^ M, respectively. The Langmuir adsorption isotherm governs the adsorption of synthetic pyrazoles on steel surfaces. DPC-1 and DPC-2 behave as mixed-type inhibitors, according to the reference [24]. The theoretical and experimental study of pyrazole pyridine and pyrazole carboxylate derivatives as corrosion inhibitors for MS surface in 15% HCl solution was conducted using different techniques. These tested compounds show a phenomenal capacity of protection against the corrosion of MS and behave as mixed corrosion protection (mixed-type adsorption). SEM and AFM analyses confirmed the existence of a protective film of the inhibitor on the MS surface [25]. Similarly, wear protection by pyrazoles has been prompted by a group of researchers who have developed new ways of using them since these derivatives as inhibitors have high chemical activity associated with high solubility in acids, with minimal environmental risk and toxicity, compared to other classes of organic compounds [26,27]. Previous studies have also examined the influence of temperature on pyrazoles in phosphoric media, as well as the effect of concentration. The results revealed that with temperature the pyrazole strength decreases, while increasing the concentration results in a decrease in current density and consequently potential efficiency reaching 97% at 10^−3^ M [28].

One of the considerations that prompted the study of pyrazoles is the fact that these derivatives are widely used as corrosion protection agents, as revealed in several literature reviews. Also, the possibility of making suitable substitutions with desired functional groups, renders pyrazoles attractive candidates for the synthesis of new corrosion inhibitors.

On this basis, we were interested in continuing our research into corrosion inhibitors. We chose to study the same pyrazolo-pyrimidine compound that was tested by our team in phosphoric acid and showed remarkable effectiveness against corrosion of 904L stainless steel [28].

The aim of this work is to extend this research by investigating the inhibitory properties of pyrazole on the corrosion of MS in 1 M HCl using electrochemical and non-electrochemical methods. The adsorption behavior of this inhibitor compound in molar hydrochloric acid is discussed. The effects of temperature and isothermal adsorption are also determined. To acquire basic knowledge on the interactions and adsorption of 1,5-diallyl-1H-pyrazolo [3,4-d]pyrimidin-4(5H)-one (PPD) on the metal surface, computer simulations (quantum chemistry linked to Monte Carlo simulation modeling) were applied. Figure S1 presents the structure of the pyrazole derivative examined in this work.

## 2. Materials and Methods

### 2.1. MS Sample and Test Electrolyte

Both electrochemical and non-electrochemical approaches were undertaken on MS, also referred to as ordinary steel and low carbon steel, which is one of the industry’s preferred materials due to its availability, physical properties, and cost. MS was applied for this investigation according to the following % composition: (C = 0.22, Mn = 0.05, Si = 0.38, S = 0.05, P = 0.09, Al = 0.01, and the rest iron). The exposed surface area of samples was 1 cm^2^, and they were mechanically abraded with different grade emery papers (120, 220, 400, 600, 800, 1500, and 2000 grade) before testing. The coupons were rinsed with distilled water, degreased with acetone, dried and kept in vacuum desiccators. The test electrolyte (1 M HCl) was prepared by diluting Analar grade 37% hydrochloric acid with ultra-pure water. All measurements were repeated, at least, three times to obtain a satisfactory reproducibility.

### 2.2. Inhibitor Elaboration

From a medium containing of 1H-pyrazolo [3,4-d]pyrimidin-4(5H)-one (0.5 g, 3.67 mmol) dissolved in DMF (30 mL) was introduced 3-bromoprop-1-ene (1.4 mL, 7.4 mmol), potassium carbonate (1.02 g, 7.4 mmol), and a catalytic amount of tetra-n-butylammonium bromide (0.1 g, 0.4 mmol) (Figure S2). The entire contents of the preparation were agitated for 48 h and the reaction monitored by thin layer chromatography. The mixture was filtered, and the solvent was separated under vacuum. Following vaporization of the solvent under low pressure, the residue obtained was chromatographed on a silica column (hexane/ethyl acetate 4:6 *v*/*v*). Recrystallization occurred in the same eluent. The obtained product was characterized by ^1^H NMR, ^13^C, IR, and mass [28].

The identification of the light yellow solid compound was carried out (56 % yield), 1H NMR (DMSOd_6_):_δ_4.59 (dd, 2H, N_5_CH_2_, ^3^J = 4.59, ^4^J = 4.57 Hz), 4.89 (dd, 2H, N_1_CH_2_, ^3^J = 4.9, ^4^J = 4.68 Hz), 4.94 (m, ^2^H, =CH_2_, ^3^J = 4.9, ^4^J = 4.6), 5.1 (m, 2H, =CH_2_, ^3^J = 5.1, ^4^J = 4.8), 5.95 (m, 1H, =CH, ^3^J = 5.93, ^4^J =5.63), 6.02 (m, 1H, =CH, ^3^J = 5.91, ^4^J = 5.71 Hz), 8.10 (s, 1H, H-3), 8.34 (s, 1H, H-6). 13C NMR (DMSO-d_6_): _δ_ 47.46 (N_5_-CH_2_), 49.56 (N_1_-CH_2_), 105.46 (C, C-3a), 117.79 (=CH_2_), 118.02 (=CH_2_), 133.88 (=CH_2_), 135.08 (CH, C-3), 151.06 (CH, C-6), 151.62 (C, C-7a), 156.60 (CO, C-4). HRMS (APPI) calcd for C_11_H_12_N_4_O (M + H) + *m*/*z*: 217.11.

### 2.3. Weight Loss (WL)

The gravimetric experiments were performed in a double glass cell equipped with a thermostatically controlled cooling condenser. The volume of the solution was 50 mL. The specimens had a rectangular shape. At the close of the trials, they were washed well with ethanol under ultrasound and then weighed. Experiments were repeated three times and the mean value of the weight loss (WL) was reported. The WL allowed us to calculate the average corrosion rate (*CR*) expressed in mg cm^−2^ h. Corrosion rate values were estimated using the following equation [29]:

(1) $$CR=\frac{Weight\;loss\;(mg)}{\mathrm{area}\;(cm^{2})\times time\;(h)}$$

From the corrosion rate, the percentage of protective effectiveness of WL experiments was calculated using the following equation [29]:

(2) $$E_{WL}=\frac{CR_{\mathrm{uninhibt}}-CR_{\mathrm{inhibt}}}{CR_{\mathrm{uninhibt}}}\times 100$$

where *CR*_uninhibit_ and *CR*_inhibit_ are the corrosion rates in the absence and in the presence of inhibitor (anti-corrosion agent), respectively.

The values of surface coverage (*θ*) were calculated by the following equation:

(3) $$\theta =\frac{E_{WL}}{100}$$

### 2.4. Electrochemical Measurements

All electrochemical measurements were made at 303 K with a thermostated water bath. The electrochemical cell had three electrodes. The reference electrode was a saturated calomel electrode (SCE). A platinum electrode was employed as an auxiliary electrode, and MS was used as a working electrode; both these electrodes had a surface area of 1 cm^2^. Electrochemical measurements included the open circuit potential (OCP), potentiodynamic polarization curves (PDP), and electrochemical impedance spectroscopy (EIS) analysis. These experiments were carried out by means of a Volta lab potentiostat. The measurements of the Tafel lines were carried out under the selected conditions mentioned above with varying content of PPD by applying the electrode potential from −800 mV to −200 mV with respect to the corrosion potential at a slew rate of 0.5 mV/s. On the other hand, EIS tests were completed in the frequency range of 100 kHz to 10 mHz at OCP with 10 points per decade, by applying an AC voltage of 10 mV peak to peak. Impedance plots are given in the Nyquist/Bode representations.

### 2.5. Computational Theoretical Studies

#### 2.5.1. Computational Method (DFT Techniques)

All chemical computations of PPD were conducted by means of the natural bond orbital (NBO) analysis through the DFT electronic structure program Gaussian 16 Rev C.01. The PPD in the isolated and neutral form and the whole complexes were minimized, calculated on the basis of B_3_LYP/6-31G(d,p) set in corrosive acid medium [30]. The elements of quantum chemistry such as *E*_HOMO_ and *E*_LUMO_, useful to elucidate the chemical descriptors of the Fe-(PPD) complex, were determined. In addition, the energy gap (∆*E*_g_), which determines the reactivity of the whole complex formed, was calculated as ∆*E*_g_ = *E*_HOMO_ − *E*_LUMO_ [30,31]. The relationship between the experimental findings and the quantum chemical descriptors for the neutral form and the complexes in different active centers are discussed in this work.

#### 2.5.2. Monte Carlo/SAA Simulation

Monte Carlo simulation coupled to simulated annealing algorithm (SAA) was carried out using three heating ramps with 15 × 10^−3^ steps per each one [32]. To get results with excellent quality, the convergence threshold was fixed at 0.015 Å, 0.5 kcal/mol.Å and 10^−3^ kcal/mol, for displacement, force, and energy, respectively. The adsorption location program involves the strategy Metropolis sampling Monte Carlo method to identify the lowest energy configuration, lowest adsorption energy, and other significant information about the energy of the system.

The sum of the deformation energy *D*_energy_ (energy of the system as long as the optimization of the geometry is completed) and of the rigid adsorption energy RAE (before the optimization of the geometry) gives the adsorption energy *E*_ads_ [30]. This second set of geometry optimization takes place when the PPD in hydrochloric acid medium is added to the surfaces of Fe (100), Fe (110) and Fe (111) under investigation (Figure S3).

The crystallographic pure iron (100), pure iron (110) and pure iron (111) planes were enlarged to (6 × 6 × 6) super-cell to offer an extensive surface for the interaction with a single inhibitor. The pure iron surface docking was used to calculate the adsorption (interaction) energies for {PPD / Fe (110), Fe (110) and Fe (110)} complexes. For electrostatic and Van der Waals interactions, the Ewald summation method, and atom-based summation method were preferred, respectively. The COMPASS force field was used and the calculations of this part were performed with the help of Adsorption Locator tools in Biovia Material Studio 2020 Dassault systems software [33,34]. The COMPASS FF was implemented to calculate intermolecular interaction energies for the PPD docked on the Fe (pure) surface in a 3D-simulation crystal structure Fe (100), Fe (110), and Fe (111) − (L_x_ = L_y_ = 35/L_z_ = 40) with periodic boundary conditions. Next, above the iron (100), iron (110) and iron (111) surfaces, a vacuum slab with a thickness of 10 Å was built. The simulation details on the procedure of Monte Carlo/SAA can be found in previous articles [35].

### 2.6. SEM Inspection

For the surface morphological examination of the uninhibited and inhibited MS samples with 10^−3^ M PPD inhibitor in 1 M HCl, scanning electron microscopy (SEM) images were recorded using the equipment provided by FEI (model: FEI-Quanta 650) with an acceleration energy of 20 kV. The tests were carried out with a 24 h immersion before proceeding with the characterization.

## 3. Results and Discussion

### 3.1. Weight Loss Measurements (WL)

The weight loss approach is a conventional method of evaluating corrosion rate. Indeed, it has been mentioned in many research studies as a powerful tool for estimating metal loss [29,36]. Table 1 lists the WL data of specimens in 1 M HCl solution at 303 K without and with various concentrations of the added organic substance.

Table 1 highlights that the corrosion process diminished in rate and the effectiveness of the PPD protector improved with rising concentrations of the compound studied (*E*_WL_ = 94% at 10^−3^ M). Such behavior of pyrazolo pyrimidine in 1 M HCl is due to the increasing surface coverage with the concentration of the compound tested. This may be explained in terms of adsorption onto the metal surface, meaning that the test compound can be adsorbed onto the metal surface through the interaction between the electron pairs of the nitrogen and oxygen atoms of the protecting agent and the metal surface. This process is facilitated by the presence of vacant low-energy orbitals in the MS atoms, as observed in the transition group metals [12].

### 3.2. OCP Measurements

The OCP is the potential of the working electrode relative to the reference electrode when no net current is circulating in the cell. Before performing potentiodynamic polarization and EIS, it is necessary to maintain the stability of the OCP [37].

**Figure 1:**
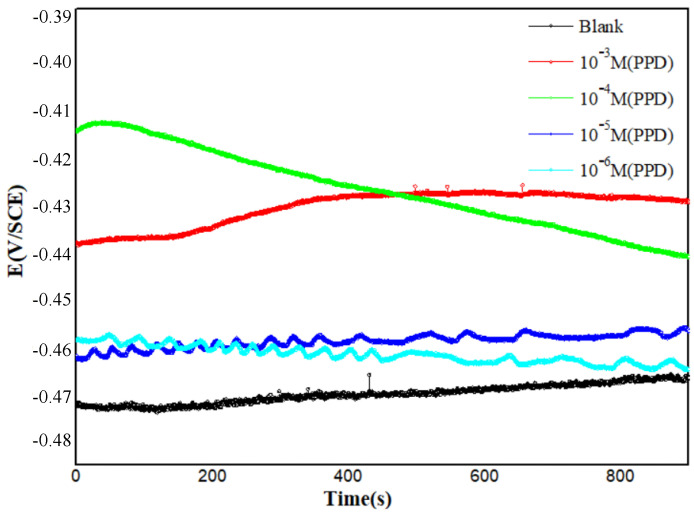
Chrono-potentiometric (zero current) curves for steel in 1 M HCl without and with different concentrations of PPD at 303 K.

Figure 1 provides the stability of the open circuit potential for all curves around at 800 s, indicating that 900 s is more than enough for the system to obtain a stable OCP [38]. The character of OCP with the presence of PPD was little changed from that without PPD. This is related to differences in surface metal activities in the inhibited and non-inhibited cases. An initial decrease in OCP suggests that the oxide film formed by the air on the electrode dissolves in the acidic medium. A later slight increase in potential may be attributed to the formation of insoluble Fe (III) oxide leading to a passive state on MS [29,39]. The addition of the studied concentrations of PPD to a 1 M HCl solution shifts the OCP to a more positive value, this displacement of which can be explained in terms of the formation of a protective layer of inhibitor on the metal surface [40]. Also, we note for the concentration of 10^−4^ M, OCP decreases rapidly towards cathodic values with time, which may be due to the dissolution of corrosion products and probably to the fact that the PPD adsorption process on the electrode surface follows slow kinetics similar to the weak PPD adsorption [40]. However, for 10^−3^ M, the OCP changes to a more positive value after 200 s and tends to stabilize after 380 s. This suggests the fast formation of a protective layer which has adsorbed on the sample surface, protecting it from attacks by the aggressive medium, and eventually improving its resistance to corrosion in an HCl solution. Therefore, it can be stated that PPD is capable of retarding the reactions that occur on the MS surface, namely, the oxidation of iron and the reduction of hydrogen ions in 1 M HCl [41].

### 3.3. Tafel Plots

**Figure 2:**
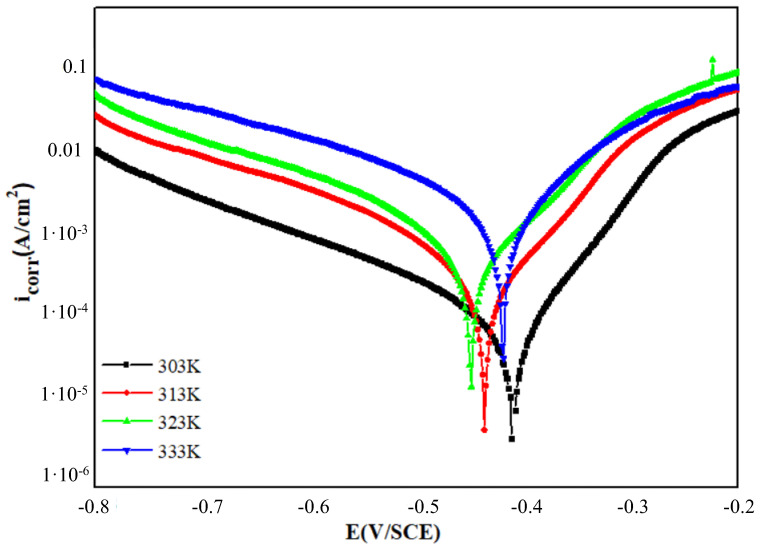
Polarization curves for (MS) in 1 M HCl with different concentrations of PPD at 303 K.

In order to better understand the effect of PPD on the electrochemical process associated with the metal corrosion phenomenon, polarization determinations were made with and without PPD at 303 K. Figure 2 depicts these curves.

The Tafel extrapolation treatment was used to obtain estimates of electrochemical parameters such as corrosion current density (*i*_corr_), corrosion potential (*E*_corr_), Tafel slope constants computed from Tafel plots (*β*_a_, −*β*_c_), and the protective efficiency acquired from potentiodynamic polarization (*E*_PDP_ %), and they are tabulated in Table 2. *E*_PDP_ was determined from Equation (4) [42]:

(4) $$E_{\mathrm{PDP}}=\frac{i_{\mathrm{corr}}^{0}-i_{\mathrm{corr}}}{i_{\mathrm{corr}}^{0}}\times 100$$

where *i*_corr_ and $i_{\mathrm{corr}}^{0}$ are the corrosion current densities with and without PPD compound studied, respectively.

It can be seen from the figure that the presence of PPD in the HCl solution caused the corrosion potential to shift towards the anodic region compared to the blank, and their value is close to that obtained in Figure 2 with almost the same trend, highlighting the inhibition capacity of PPD in acidic media.

Besides, chronopotentiometry proves that the OCP of the optimal PPD inhibitor shifted to the positive domain, which shows that the action of PPD on the anodic reaction is more pronounced compared to the cathodic reaction. However, the largest potential shift was 45 mV, significantly below 85 mV, which indicates that PPD is a mixed-type inhibitor [8]. Table 2 shows that the cathodic and anodic Tafel slopes change with PPD, the trend being more pronounced for the anodic slope, supporting that the PPD exhibits an anodic predominance [43]. In addition, analysis of the data shows that the PPD compound also decreased the corrosion current densities to lower values. So, these results reflect a decrease in dissolved mobile metal ions due to the blocking of active sites on the MS surface by adsorbed PPD molecules. Both branches are shifted to lower values of *i*_corr_.

The variation in *β*_c_ values after introducing PPD revealed that the hydrogen reduction reaction is also influenced. This may probably be the result of the coverage of the active surface sites by the adsorption of inhibitor molecules [44,45]. Likewise, it was observed that the *β*_a_ values varied in the medium containing PPD compound, implying that PPD under study was first adsorbed and obstructed the interface by simply blocking the reaction sites on the MS surface.

Chaitra et al. suggested that when the slopes *β*_a_ and *β*_c_ values show a variable trend, it indicates that several mechanisms take part in the corrosion inhibition and not only the adsorption effect, namely, the involvement of other species/anions present in the corrosive solution [45].

It seems that in the presence of PPD, the barrier film formed on the MS and the corrosion rate is limited [46]. Nevertheless, for a potential higher than −300 mV, the presence of PPD did not affect the characteristics of the current as a function of the potential in the anodic region (Figure 2). This latter may be referred to as the desorption potential. This phenomenon can be explained by the equality of the adsorption rate of the PPD compound and the oxidation rate of the metal leading to a desorption of the PPD molecule from the MS surface [47].

We can report that the value of the current density decreases with increasing PPD concentration, while the effectiveness of protection increases. The PPD is more efficient at 10^−3^ M and reaches 94 %, which follows the conclusions drawn by the gravimetric method.

### 3.4. EIS Methods

**Figure 3:**
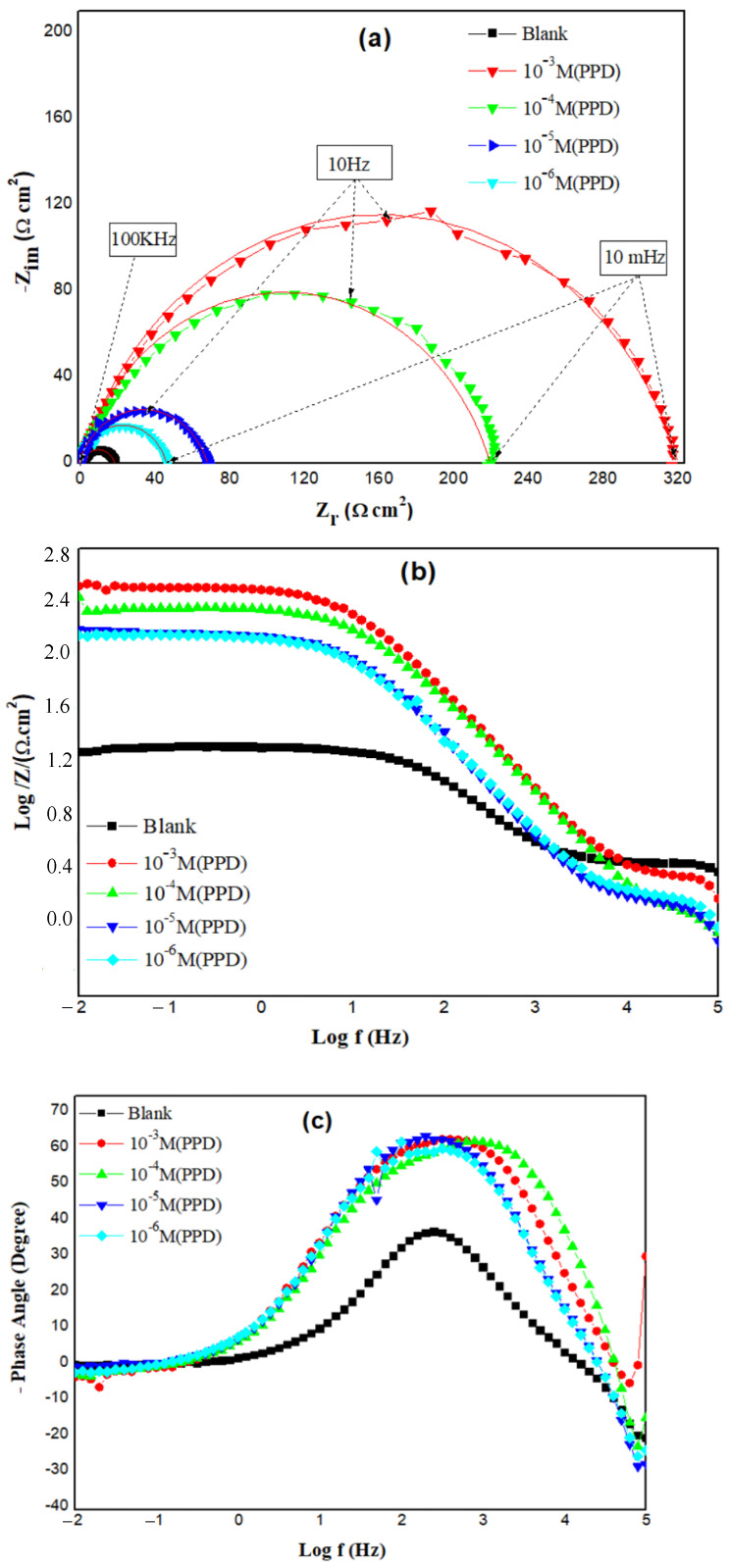
(**a**) Nyquist plots, (**b**) Bode-module plots, (**c**) Bode-phase plots of MS/HCl electrochemical system containing various PPD concentrations.

Nyquist and Bode plots are seen in Figure 3. The Nyquist plots for the inhibited and uninhibited cases present a capacitive loop, indicating that the acid dissolution of steel involves a single charge transfer mechanism [28].

The size of the semicircle in the Nyquist plots increases with PPD concentration. This finding implies that PPD may form an effective interface barrier against the charge transfer process. Besides, we notice that the shape for the Nyquist plots of the inhibited and uninhibited systems is similar. The Bode modulus patterns have two ranges: the first at high frequency is related to the solution resistance (*R*_s_), the other at low frequency is the charge transfer resistance (*R*_ct_)_._ The module of the impedance |Z| at low frequency that is linked to (*R*_ct_) increases with the PPD concentration.

Only one time constant is detected in the phase graphs, and their range widens with inhibitor concentration, resulting in a decrease in the metal dissolution rate. All these comments confirm that PPD has the ability to prevent and control corrosion by adsorption of its molecules at the interface [48].

Equivalent electrical circuits were employed to process the impedance curves. An analogous circuit matched the Nyquist and Bode graphs using (*EC-Lab*) software (see Figure S4), which has been used previously by other authors to simulate the iron–acid contact [49]. This circuit consists of a charge transfer resistance (*R*_ct_) in parallel with a double-layer capacitance (*C*_dl_), both of which are in series with the solution resistance (*R*_s_). In place of (*C*_dl_), a constant phase element (CPE) was introduced in the analogous circuit, which is connected with the heterogeneity of the MS due to surface roughness and the adsorption of PPD molecules [22]. The introduction of such a CPE instead of a capacitor explains the deviations from the ideal dielectric behavior. The impedance, Z, of the CPE is given by the formula below:

(5) $$Z_{CPE}=Q^{-1}{\left(j\omega \right)}^{-n}$$

Considering that *Q* is the CPE modulus, *j* is the imaginary unit, *ω* is angular frequency and *n* an exponential factor which defines the surface irregularity due to impurities such as corrosion products, adsorption of inhibitor, and surface roughness. The *n* values are often varied between 0 and 1.

The corresponding fit results obtained from the EIS plots for the PPD inhibitor at different concentrations are summarized in Table 3. The double-layer capacitances, *C*_dl_, for a circuit comprising a CPE were determined by utilizing this formula [50]:

(6) $$C_{dl}={\left(QR_{ct}^{1-n}\right)}^{1-n}$$

Based on the charge transfer resistance (*R*_ct_), it is possible to determine the protection efficiency (*E*_EIS_ %) according to the equation below:

(7) $$E_{EIS}\%=\frac{R_{ct}-R_{ct}^{0}}{R_{ct}}\times 100$$

where the resistances of charge transfer with and without the protective substance (PPD) are $R_{\mathrm{ct}}^{0}$ and $R_{\mathrm{ct}}$, respectively.

According to Table 3, the values of *R*_ct_ and protective efficiency are more affected by the increase in PPD concentration. Indeed, *R*_ct_ values significantly increase with PPD and achieve 319 Ω cm^2^ at 10^−3^ M. This performance is related to the creation of a barrier layer at the metal/solution interface which strongly inhibits local corrosion induced by chloride ions. The results showed that PPD molecules, instead of water molecules, act on the surface of the MS by adsorption at the steel/electrolyte interface as reported by some scientists [50].

Remarkably, the *n* values obtained for the non-inhibited solution are close to that of the inhibited one and simply indicate the surface inhomogeneity due to the adsorption of PPD molecules. The capacity values of the double layer *C*_dl_ as well as the coefficient *Q*, calculated in the presence of PPD, are inferior to those of the blank medium.

The action of the PPD derivative in the electrochemical system studied can affect the behavior of the electrochemical reaction involved in the mechanism of the corrosion process, on the one hand by modifying the potential gradient in the double layer due to the change of the dielectric constant of the medium between MS and HCl. On the other hand, it may reduce the interaction zone and raise the distance between the different elements participating in the reaction due to the inclusion of the organic compound between the MS surface and the Helmholtz plane (i.e., center of positive charges, which resides at a fixed distance from the metal due to the water molecules between the metal surface and the ions), which results in more PPD inhibitor adsorption. This leads to an increase in thickness of the inhibitor adsorbed layer, which in turn results in a decrease in the double-layer capacitance [51].

The maximum inhibition efficiency is 94%, obtained when the concentration of the PPD solution is 10^−3^ M. This result can be explained by the adsorption of more PPD molecules at higher concentrations, thereby better protecting the surface of the MS from the aggressive attack of the solution [49,52].

### 3.5. Temperature Effect

**Figure 4:**
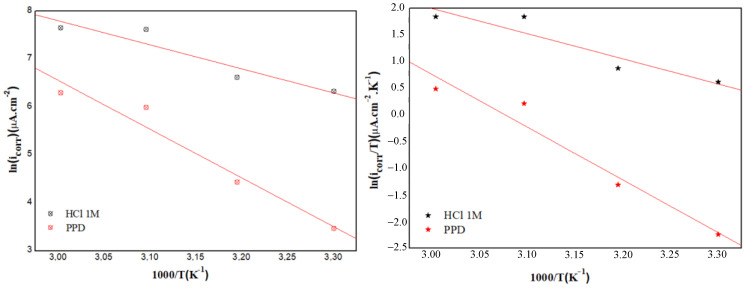
Arrhenius and transition Arrhenius plots of MS in 1.0 M HCl containing the optimal concentration of 10^−3^ M PPD.

Potentiodynamic polarization tests were also made in the temperature domain of 303 to 333 K, as part of the evaluation of PPD adsorption and activation variables of the MS oxidation process in acidic solution without and with 10^−3^ M PPD. The results are summarized in Figure 4, Table 4 and Table S1.

Table S1 illustrates that the inhibition efficiency decreases with increasing temperature, which can be attributed to a possible shift in the adsorption–desorption equilibrium toward desorption of the adsorbed PPD inhibitor at higher temperature, leading to the surface area of the metal to be exposed to the acidic environment. The percentage of protective effectiveness values gradually dropped from 94% at 303 K to 74 % at 333 K for a 10^−3^ M of PPD.

Thus, in examining the effect of temperature on the corrosion process in the presence of the 10^−3^ M PPD, the use of the Arrhenius equation is very useful.

So, the activation parameters which comprise the activation energy (*E*_a_), the activation enthalpy (Δ*H*_a_), and the activation entropy (Δ*S*_a_) were predicted by Arrhenius’ law and state relations transition [53] given by Equations (8) and (9), respectively:

(8) $$i_{\mathrm{corr}}=A\exp\frac{E_{a}}{RT}$$

(9) $$i_{\mathrm{corr}}=\frac{RT}{Nh}\exp\frac{\Delta S_{a}}{RT}\exp\frac{\left(-\Delta H_{a}\right)}{RT}$$

where *A* is the frequency factor, *N* is Avogadro’s number, *h* is Planck’s constant and R is the universal gas constant.

From Figure 4, the MS activation energy for the blank solution and for the 10^−3^ M PPD-added media is calculated using the ln (*i*_corr_) vs. 1000/T representation, while the values of the components Δ*H*_a_ and Δ*S*_a_ are calculated via ln (*i*_corr_/*T*) vs. 1000/*T* plots. These results are grouped in Table 4.

Indeed, the acquired values of (*E*_a_) in the presence of 10^−3^ M PPD are much higher (84.57 kJ mol^−1^) than that of the uninhibited solution (*E*_a_ = 41.73 kJ/mol) while increasing the temperature, revealing that PPD adsorbs within the 303 to 333 K range with an electrostatic character process on the MS surface [54,55]. This conclusion is denoted by the decrease of the inhibition efficiency with increasing temperature (Table S1). The higher *E*_a_ values suggests that the energy barrier for the corrosion reaction increases in the presence of PPD molecules, thereby reducing the corrosion rate. Therefore, all these findings agree with what has been reported in previous studies [54,56].

The positive values of (Δ*H*_a_) show the nature of the dissolution of the MS and reflect an endothermic activation process. However, the increase of the activation enthalpy with the PPD concentration is associated with a reduced dissolution of the metal [57]. As seen in Table 4, the difference between *E*_a_ and Δ*H*_a_ is positive for both inhibited and non-inhibited electrolytes, and for both cases *E*_a_ > Δ*H*_a_ by a value that is approximately close to R*T*. As reported in the literature, from a thermodynamic and kinetic point of view, the following equation can be defined as [58]:

(10) $$E_{a}-\Delta H_{a}≃R\times T$$

It was found that the value of the entropy (Δ*S*_a_) for the reference solution is negative, while that for the inhibited solution is positive. However, the activation complex characterizing the rate-determining step, which represents an association rather than dissociation step, has been justified in previous work by the substitution process [59]. The high value of (Δ*S*_a_) is interpreted by the increase in disorder due to the presence of more water molecules, which can be desorbed from the surface of the MS by the inhibitor molecule [60].

### 3.6. Adsorption Consideration

The most critical step in the inhibition mechanism of organic compounds is their adsorption onto the metal surface. The adsorption phenomenon can be studied with the help of adsorption isotherms, which can then be useful to determine the adsorption type, i.e., physical adsorption, chemical adsorption, or mixed adsorption [61].

**Figure 5:**
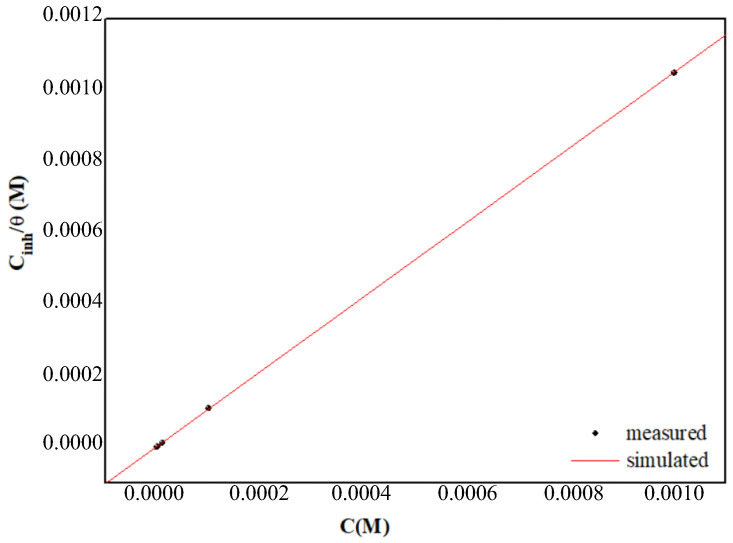
Langmuir adsorption of PPD on (MS) surface in molar HCl.

The adsorption of PPD on the metallic surface can be evaluated by fitting the experimental data obtained from the polarization curves for the various adsorption isotherms, such as Frumkin, Langmuir and Temkin. The closest fit was found with the Langmuir adsorption isotherm, as illustrated in Figure 5 and described by the formula [62]:

(11) $$\frac{C_{\mathrm{inh}}}{\theta }=\frac{1}{K_{\mathrm{ads}}}+C_{\mathrm{inh}}$$

knowing that, *C*_inh_ is the concentration of PPD, *K*_ads_ is the adsorption equilibrium constant, and *θ* is the surface coverage.

The values of the adsorption coefficient (*K*_ads_), determined by extrapolation of the lines obtained in the graph, were then used to access the value of the standard free energy of adsorption (∆$G_{\mathrm{ads}}^{0}$) represented by the following formula [63]:

(12) $$\Delta G_{\mathrm{ads}}^{0}=-RTln(K_{\mathrm{ads}}55.5)$$

where R is the gas constant (8.314 J K^−1^mol^−1^), *T* the absolute temperature in Kelvin, and the value 55.5 is the concentration of water in the solution, in mol L^−1^.

Plotting *C*_inh_/*θ* versus *C*_inh_ provides a straight line (see Figure 5). Also, both the linear correlation coefficient (r^2^) and the slope are close to 1 (Table 5), indicating that the adsorption of PPD on the MS surface is in accordance with the Langmuir adsorption isotherm. The larger magnitudes of *K*_ads_ reflect the strong adsorption of the PPD chemical species on the material surface.

Moreover, ∆$G_{\mathrm{ads}}^{0}$ was determined as −44.5 kJ mol^−1^ for PPD. The ∆$G_{\mathrm{ads}}^{0}$ value of adsorption indicates the strong interaction between inhibitor molecules and the metal surface [64].

Generally, standard free energy values of −20 kJ mol^−1^ or less negative are associated with an electrostatic interaction between charged molecules and charged metal surface (physical adsorption); those of −40 kJ mol^−1^ or more negative involves charge sharing or transfer from the inhibitor molecules to the metal surface to form a coordinate covalent bond (chemical adsorption) [8,65,66].

Therefore, the experimental results such as the thermodynamic and isotherm part of the present study reveal that the adsorption mechanism adopted by the inhibitor is mainly due to the interaction of the active sites on the inhibitor molecule with the ionized atoms on the MS surface as reported in the literature. In other words, these interactions can be a combination of both physical and chemical, as in our case. Indeed, the action may involve on the one hand, the interaction between the pairs of non-bonding electrons on the heteroatoms with the unoccupied (d) orbitals of the Fe atoms, and therefore responsible for the chemical adsorption. On the other hand, in acidic medium, the protonation of PPD is achieved via nitrogen and oxygen atoms, also, the interaction occurs between negatively charged Cl^−^ ions on the surface of MS and positively charged inhibitor molecules which may adsorb through electrostatic interactions. Moreover, the π-electron clouds on the aromatic ring also participate in a donor–acceptor interaction (retro-donation) with the ionized Fe atoms on the surface [64,65,66].

### 3.7. Metal Surface Inspection

**Figure 6:**
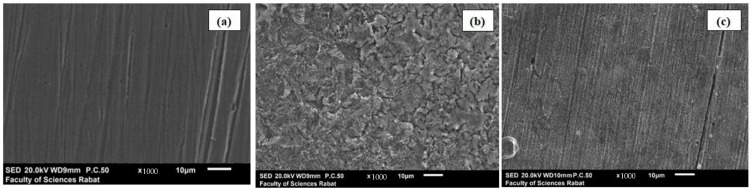
SEM micrographs: (**a**) metallic surface after being polished, (**b**) metallic surface after immersion in 1 M HCl alone and (**c**) metallic surface after immersion in 1M HCl + 10^−3^ M PPD.

SEM observations of MS specimens prior to and after dipping in 1 M HCl solution alone, and with 10^−3^ M PPD, are illustrated in Figure 6a–c, respectively.

The morphology of the specimen before immersion (Figure 6a) is very smooth and exhibits no growth of corrosion. The SEM image taken after dipping the specimen in HCl medium for 24 h depicts that a rust layer has been created with a fine plate-like structure covering the entire metallic surface exposed (Figure 6b).

On the other hand, Figure 6c shows that the surface of the inhibited MS is covered with a smooth layer of the added organic product, in addition to some polishing scratches. This explains that the metal is protected from the corrosive environment by reducing the active corrosion centers. This observation shows that the inhibition is due to the formation of a stable protective layer on the surface of the MS by adsorption of the PPD, which limits the access of the electrolyte to the metal surface.

As such, the SEM representations are in close agreement with the various findings from the weight loss and electrochemical investigations.

### 3.8. Molecular Structural Reactivity Examination

#### 3.8.1. Behavior of the Chemical Reactivity of Neutral and Protonated Forms—Global Reactivity

The progress of molecular electronics requires efficient and reliable computational procedures for the simulation of the atomic structures of real devices, including the prediction of their electronic properties [67,68]. Density function theory (DFT) has been very successful in chemistry and solid-state physics [69], and it offers the possibility to fulfil these roles.

In this work, the minimized geometry of PPD specie in the corrosive medium used by the DFT/BLYP method with a 6-31G(d,p) basis set is approximately planar.

However, the adsorption on the metal area and donation of Pi (π) systems can become easy via the (diallyl-pyrazole-pyrimidin-one) rings, as well as the unbound e-pair of heteroatoms (=N- and -O-) of the PPD molecule in the vacant 3d-iron orbital [36]. Figure S5 shows a view of the energy-minimized 3D-geometry of PPD specie in the aqueous phase calculated using B3LYP/6-31G(d,p).

**Figure 7:**
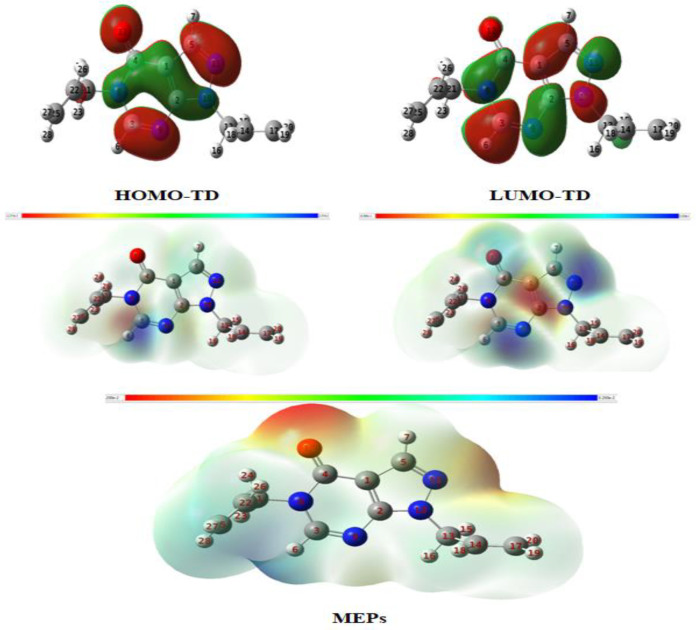
Minimized molecular structure, FMO, and MEP plots for PPD specie in the aqueous phase using the DFT/BLYP method with 6-31G(d,p) basis set.

Figure 7 shows that (HO/LU) MO are clearly highly delocalized in the (Pyrazole-pyrimidine one) ring of PPD. The Kohn–Sham FMO electron density contour plots depend on the structure and location of the electron density HOMO in the PPD, which is found mainly distributed on heteroatoms with delocalized aspect, pointing out that the later are the favorite adsorption active sites (X: O_15_ and N_16_). Meanwhile, it has been signaled that the best experimentally tested inhibitor is the one that marks its contribution by the HOMO that is found in the ring (benzene thiazine one) as in our case of PPD. By means of the DFT / BLYP approach with the LANL_2_DZ basis set as displayed in Figure 7, (HO / LU) MO of a geometry in which a single one of (Fe by Fe^2+^, Fe^3+^ conversion) is simultaneously interacting with (X: O_15_. S_14_ and N_16_).

Binding energy (∆*E*_Binding_) or the interaction energy (in absolute value) between the Fe^2+^ iron surface and inhibitor molecule were evaluated according to Equation (13):

(13) $$\Delta E_{\mathrm{Binding}}=-E_{\mathrm{complex}}+E_{\mathrm{Fe}}+E_{\mathrm{inh}}$$

**Figure 8:**
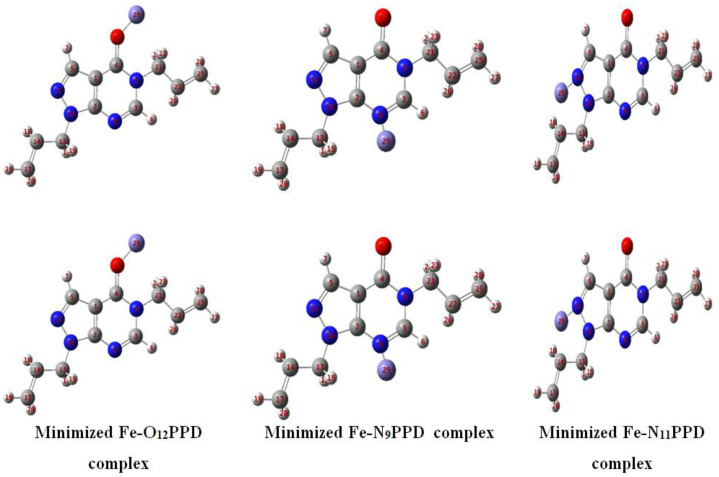
Energy minimized structures of Fe−O_12_PPD, Fe−N_9_PPD, and Fe−N_11_PPD complexes calculated using B_3_LYP/6-31G(d,p).

Figure 8 shows the energy-minimized structures of Fe−O_12_PPD, Fe−N_9_PPD, and Fe−N_11_PPD complexes calculated using B_3_LYP/6-31G(d,p).

**Figure 9:**
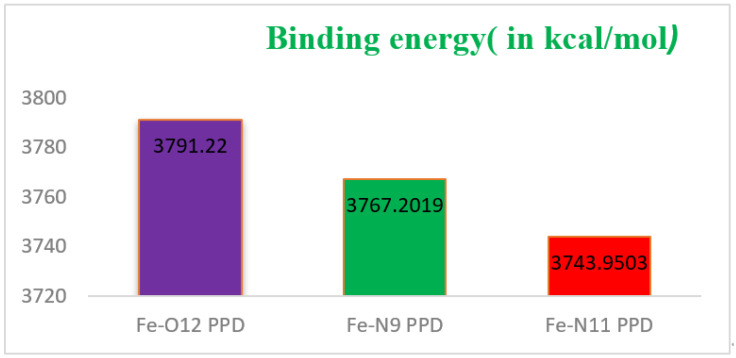
Binding energy or (interaction energy in absolute value) for Fe−X_j_ (PPD) in different active (Xj: O_12_, N_9_ and N_11_) centers.

The binding energy (interaction energy in absolute value) for Fe−Xj (Xj: O_12_, N_9,_ and N_11_) (PPD) complexes in the three active sites, is presented in Figure 9 and computed by the mean of the DFT/B_3_LYP/LANL_2_DZ scale.

Adsorption energy is a useful tool showing the corrosion inhibition activities of inhibitors during the simulation process. This energy reflects the power of the inhibitor–metal surface interaction. Effective corrosion inhibitors have more negative adsorption energy values and can adsorb more easily on metal surfaces.

For the three complexes, the ∆*E*_Binding_ (in kcal mol^−1^) values are found to be 3791.22 for Fe−O_12_ (PPD) complex, 3767.20 for Fe−N_9_ (PPD) complex, and 3743.95 for Fe−N_11_ (PPD). The strength and stability of the adsorption complex is stated by the significant higher value of the differential binding energy.

The highest value of (∆*E*_Binding_ (∆*E*_Binding_ > 10^4^ kcal/mol) affirms that the adsorption ability of PPD in the tested solution on the iron surface (percentage of protection is large *(E%*)) is spontaneous and stable. Also, the larger positive values of ∆*E*_Binding_ can be attributed to the strong adsorption for the Fe^2+^−PPD complex (attractive force) [70].

Chemical reactivity is a dependence of the interaction between the (LUMO) and (HOMO) energy levels for the Fe−inhibitor system based on the FMO theory [71].

The *E*_HOMO_ is a designer of quantum chemistry (QCDs) that is coupled with the electron donating capacity of the molecule. It is commonly associated with high experimental inhibition [69].

**Figure 10:**
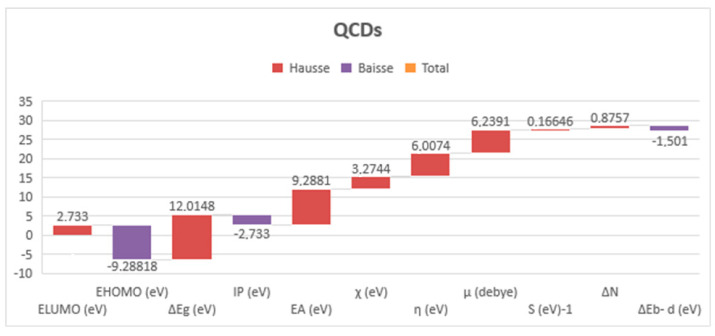
Quantum chemical descriptors for PPD used by DFT/B_3_LYP/6-31G basis set.

The interaction energy for Fe−(PPD) was found to enhance with lowering *E*_LUMO_ and raising *E*_HOMO_. Hence, the *E*_HOMO_ and *E*_LUMO_ for steel are checked against the calculated value for PPD to detect the kind of interaction [72]. All calculated QCDs are given in Figure 10 and $\Delta E_{g}=E_{\mathrm{LUMO}}^{\mathrm{inh}}-E_{\mathrm{HOMO}}^{Fe}(eV)$ for the Fe–PPD complex are presented in Table 6.

Hence, $E_{\mathrm{HOMO}}^{Fe}$ = −7.9024 eV and $E_{\mathrm{LUMO}}^{Fe}$ = −0.151 eV/$E_{\mathrm{HOMO}}^{\mathrm{inh}}$ = −9.2818 eV and $E_{\mathrm{LUMO}}^{\mathrm{inh}}$ = +2.733 eV.

From Table 6, it can be concluded that the PPD acts as a cathodic inhibitor because the iron atom [Ar] 3d^6^4s^2^ will act as a Lewis base (electron-pair donor) while the inhibitor PPD molecule acts as a Lewis acid (electron-pair acceptor). So, to react with the LUMO orbital of PPD, the Fe atom [Ar] 3d^6^4s^2^ will access the HOMO. The iron surface interaction with the PPD interface will have some ionic properties as the value of the $E_{\mathrm{LUMO}}^{\mathrm{inh}}$ − $E_{\mathrm{HOMO}}^{\mathrm{Fe}}$(eV) approximates 9 eV [73,74], whereas the most powerful kind of covalent bonding can be provided if the bandgap $E_{\mathrm{LUMO}}^{\mathrm{inh}}$ − $E_{\mathrm{HOMO}}^{\mathrm{Fe}}$ (eV) energy value is very small [73].

#### 3.8.2. Local Indices (Fukui Indices)

**Figure 11:**
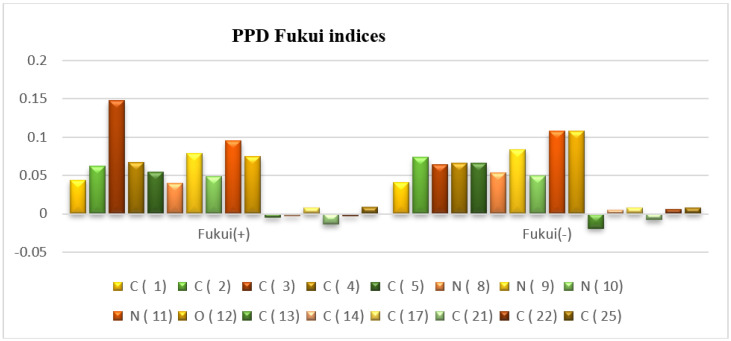
The Fukui function for nucleophilic attack Fukui (+) and electrophilic attack Fukui (−) for PPD.

To identify the local reactivity descriptor of the PPD, Fukui Indices (FIs) are necessary. In the present study, FIs were determined by the DFT method with the B_3_LYP/6-31G basis set. Following the literature, Fukui (+) denotes the higher value by computing the change of electrons distribution of the molecule on the active centers when it gains one more (*e*^−^), while Fukui (−) reflects the change of electron density of the protective molecule on the active centers when this one loses one (*e*^−^). The nucleophilic Fukui (+) attack, as well as the electrophilic Fukui (−) attack, of PPD were attained in liquid phase (water). The corresponding values for PPD in neutral state are reported in Figure 11.

As disclosed in Figure 11, it can be said that in PPD, the heteroatoms N_8_, N_9_, N_10_, N_11_, and O_12_ that have lone pairs that can react with nucleophiles possess Fukui (+) of +0.04, +0.079, +0.049, +0.091 and +0.075, respectively, and those that can react with electrophiles possess Fukui (−) of +0.054, +0.084, +0.084, +0.05, +1.108 and +0.108, respectively. Moreover, the four atoms C_1_, C_2_, C_3_ and C_4_ are more susceptible to be attacked by electrophilic and nucleophilic species.

Consequently, the molecule (PPD) acts with almost the same number of electrophiles (Lewis acid) as nucleophiles (Lewis base). This fact (by the DFT method) justifies the high efficiency of PPD.

#### 3.8.3. Monte Carlo Simulations (MCT)

**Figure 12:**
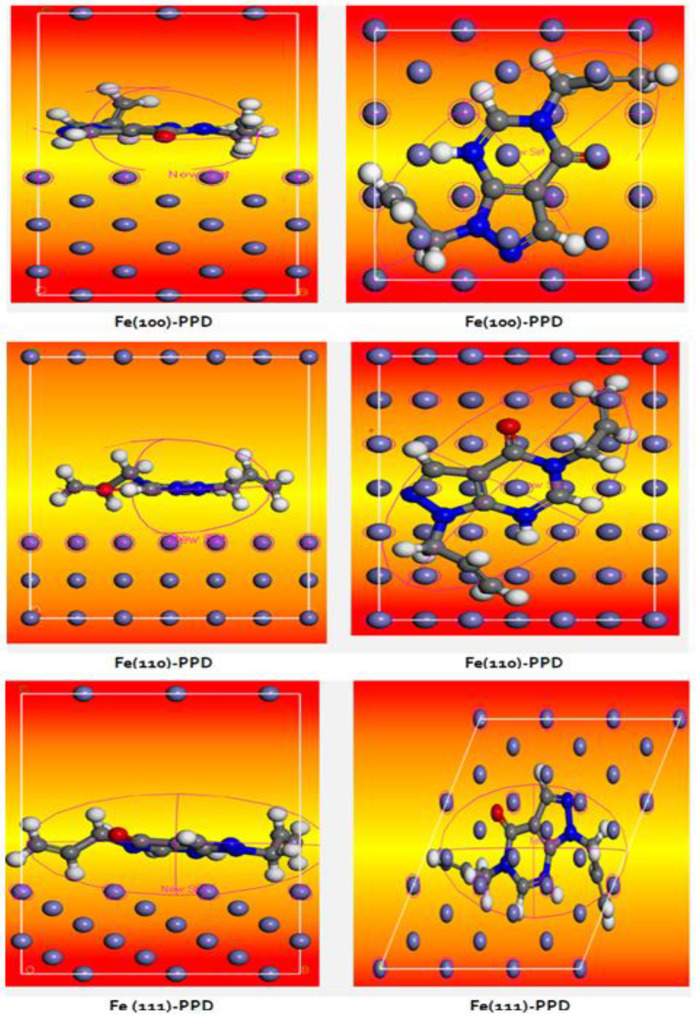
Representative snapshots of the top and side views the PPD/Fe (100), Fe (110) and Fe (111).

The most stable adsorption for the three PPD/Fe (100), Fe (110) and Fe (111) complexes is shown in Figure 12 (side and top snapshots). The adsorption patterns depict that PPD adsorbs at different sites on the iron surface. The flat adsorption pattern of PPD on Fe (100) and Fe (111) surfaces and the tilted adsorption pattern of PPD on the Fe (110) surface are in agreement with the literature [72].

Adsorption energy is a useful tool showing the corrosion inhibition activity of inhibitors during the simulation process. More negative metal inhibitor interaction energy values represent the power of the interaction between inhibitor molecules and the metal surface. The adsorption energies (∆*E*_adsorption_) for {PPD/Fe (100), Fe (110) and Fe (111)} complexes are shown in Table 7. The lowest ∆*E*_adsorption_ (in kcal mol^−1^) (in absolute value) of −135.03 was found for the Fe (100), while the higher ∆*E*_adsorption_ value of −151.08 was found for the Fe (110). The sequence of reactivity was established by scanning the lowest energy for {PPD/Fe (100), Fe (110) and Fe (111)} complexes. The interaction with the lowest ∆*E*_adsorption_ value illustrates that the reaction is more likely to occur as compared to a molecule with greater energy. Within the framework of calculated adsorption energies, we can write the corrosion inhibition efficiency order for PPD on each surface as follows: Fe (100) > Fe (110) > Fe (111). The calculations yielded negative adsorption energies, implying that all interactions were spontaneous; these readings are in close accordance with the actual experimental research for transformer corrosion [73].

Additionally, the deformation or strain energy (*D*_energy_) of −41.33, −40.08, and −39.87 kcal/mol was defined for the adsorption of PPD on Fe (110), Fe (111), and Fe (100) surfaces, respectively (see Table 7).

The Fe (110) surface was proved to be more vulnerable to deformation than both Fe (111) and Fe (100) surfaces. Indeed, these deformation energies achieved indicate a weak participation in the corrosion of the tested metallic surfaces.

The bond distance of the heteroatom from the PPD molecule to the nearest iron steel surfaces atom is given in Table 8. The shortest bond distance of PPD is of the order of *d* = 3.55 Å, indicating a strong chemical bond formation for (PPD)–iron surface complexes (combination of the chemisorption and the physisorption).

Thus, the surface of Fe (110) has the narrowest binding distance of 3.351 Å, while the bonding distance at the Fe (111) and Fe (100) surfaces was proved to be 3.879 Å and 3.479 Å, respectively. These outcomes may imply that processes such as passivation or thin films ought to be applicable to Fe (110) to minimize the possibility of PPD interaction with the surface.

## 4. Conclusions

The inhibition effects of pyrazolo-pyrimidine derivatives on steel in 1M HCl solution were studied at various concentrations. The experimental and theoretical results received lead to the following conclusions:

- The achieved WL loss results obtained showed that PPD greatly inhibited corrosion of MS in an aggressive acid environment.
- Polarization studies revealed that PPD acted as mixed-type inhibitor. EIS measurements suggested that the PPD inhibits corrosion by adsorbing at the interface and *η%* tended to increase with increasing the inhibitor concentrations, reaching its maximum value of 94% at 10^−3^ M.
- PPD was proven to be an effective corrosion inhibitor and obeyed the Langmuir adsorption isotherm.
- Scanning electron microscopy (SEM) confirmed the presence of a protective layer formed on the MS surface, limiting the access of the aggressive solution to the metal surface.
- DFT parameters showed good agreement with the experimental results. MD simulation methods have shown that PPD adsorb to the metal surface in a flat or tilted adsorption pattern, which is consistent with the literature.

## Figures and Tables

**Table 1:** Corrosion ratio and the % of protective effect on specimens following 6 h of exposure with and without PPD.

*C* (M)	*CR* (mg cm^−2^ h^−1^)	*E*_WL_ (%)	*θ*
Blank	0.600	−−	−−
10^−3^	0.037	94	0.94
10^−4^	0.062	90	0.90
10^−5^	0.142	76	0.76
10^−6^	0.252	58	0.58

**Table 2:** Tafel polarization parameters for steel/HCl electrochemical system without and with various PPD concentrations.

Inhibitor	*C* (M)	*E*_corr_ (mV vs. SCE)	*β*_a_ (mV dec^−1^)	−*β*_c_ (mV dec^−1^)	*i*_corr_ (µA cm^−2^)	*θ*	*E*_PDP_ (%)
1 M HCl	Blank	−459 ± 31	164 ± 17	92 ± 3	560 ± 13	−−	−−
PPD	10^−3^	−409 ± 49	63 ± 2	132 ± 14	32 ± 6	0.94	94
	10^−4^	−424 ± 33	41 ± 4	119 ± 16	58 ± 2	0.90	90
	10^−5^	−440 ± 16	75 ± 10	102 ± 19	135 ± 6	0.76	76
	10^−6^	−442 ± 47	71 ± 4	122 ± 4	191 ± 14	0.66	66

**Table 3:** Impedance parameters for MS/ HCl system without and with different PPD concentrations.

*C* (M)	*R*_s_ (Ω cm^2^)	*R*_ct_ (Ω cm^2^)	*Q* (µF S^n−1^)	*C*_dl_ (µF cm^−2^)	*n*	*E*_EIS_ (%)
Blank	1.16	17 ± 0.06	462 ± 2.09	138 ± 1.03	0.800 ± 0.05	−−
10^−3^	1.728	319 ± 1.09	109.2 ± 0.04	46.44 ± 0.09	0.796 ± 0.02	94
10^−4^	0.9008	219.7 ± 2.07	121.4 ± 1.08	48.23 ± 1.05	0.797 ± 0.01	92
10^−5^	0.9987	78.4 ± 2.01	254 ± 2.02	115.9 ± 0.08	0.889 ± 0.09	78
10^−6^	1.176	54 ± 3.05	286.2 ± 1.08	126.1 ± 2.03	0.790 ± 0.06	68

**Table 4:** Activation parameters for MS in the tested solution with and without PPD derived from the polarization analysis.

*C*_PPD_ (M)	*E*_a_ (kJ mol^−1^)	Δ*H*_a_ (kJ mol^−1^)	Δ*S*_a_ (kJ mol^−1^)	*E*_a_−Δ*H*_a_ (kJ mol^−1^)
Blank	41.73	39.09	−63.71	2.64
10^−3^ M PPD	84.57	81.93	54.49	2.64

**Table 5:** Parameters of (*C*/*θ* vs. *C*) and (∆$G_{ads}^{0}$) for the electrochemical system studied.

Compound Tested	r^2^	Slope	*K*_ads_ (10^5^ Μ^−1^)	∆$G_{ads}^{0}$ (kJ mol^−1^)
PPD	0.9999	1.049	8.30	−44.5

**Table 6:** *E*_HOMO_−*E*_LUMO_ interactions for MS–(PPD) complex.

Inhibitor	$\left|E_{LUMO}^{inh}-E_{HOMO}^{Fe}|(\mathrm{eV}\right)$	$\left|E_{LUMO}^{Fe}-E_{HOMO}^{inh}|(\mathrm{eV}\right)$
PPD	9.1308	10.6354

**Table 7:** ∆*E*_adsorption_ and *D*_energy_ for {PPD/Fe (100), Fe (110), and Fe (111)} interfaces.

Complexes	∆*E*_adsorption_ (kcal mol^−^^1^)	*D*_energy_ (kcal mol^−^^1^)
Fe (110)−(PPD)	−151.08	−41.33
Fe (111)−(PPD)	−135.68	−40.08
Fe (100)−(PPD)	−135.03	−39.87

**Table 8:** The bond distance for {PPD/Fe (100), Fe (110) and Fe (111)} interfaces.

Complexes	Bond Distances
Fe (110)−(PPD)	3.351 Å
Fe (111)−(PPD)	3.879 Å
Fe (100)−(PPD)	3.479 Å

## Data Availability

Not applicable.

## Supplementary Materials

The following supporting information can be downloaded at: https://www.mdpi.com/article/10.3390/ma16020678/s1, Figure S1: 1,5-diallyl-1H-pyrazolo [3,4-d]pyrimidin-4(5H)-one; Figure S2: reaction for the synthesis of 1,5-diallyl-1H-pyrazolo [3,4-d]pyrimidin-4(5H)-one; Figure S3: side view of the Fe (100), (110), (111), surface models before the Monte Carlo Simulations; Figure S4: electrochemical equivalent circuit used to fit the impedance spectra; Figure S5: view of the energy-minimized 3D-geometry of PPD specie in the aqueous phase calculated using B3LYP/6-31G(d,p); Table S1: potentiodynamic parameters of MS in 1M HCl alone, and with 10^−3^ M PPD, at different temperatures.
