# Supplementary material for: Experimental and Theoretical Tests on the Corrosion Protection of Mild Steel in Hydrochloric Acid Environment by the Use of Pyrazole Derivative
Source: Materials (Basel). 2023 Jan 10;16(2):678. doi: 10.3390/ma16020678 (PMC9862199; doi:10.3390/ma16020678)
Supplement: Supplementary file 1 [file materials-16-00678-s001.zip › materials-2100052-supplementary.pdf]

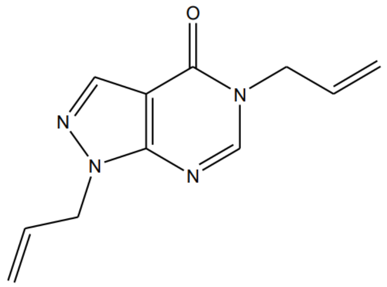

Figure S1 : 1,5-diallyl-1H-pyrazol o[3,4-d]pyrimidin-4(5H)-one

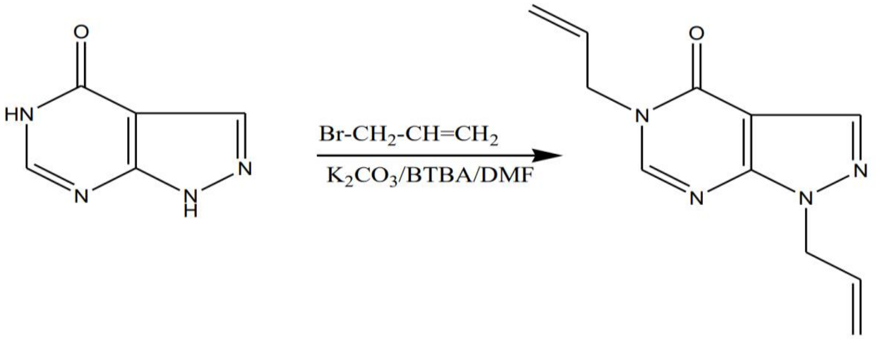

Figure S2 : Reaction for the synthesis of 1,5-diallyl-1H-pyrazol o[3,4-d]pyrimidin-4(5H)-one

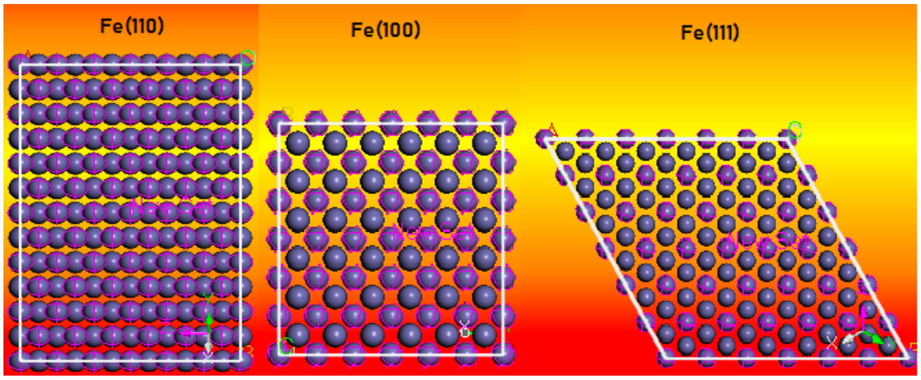

Figure S3: Side view of the Fe (100), (110), (111), surface models before the Monte Carlo Simulations

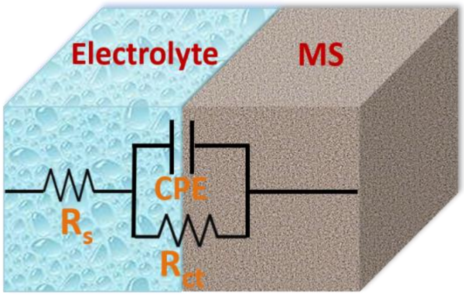

Figure S4: The electrochemical equivalent circuit used to fit the impedance spectra

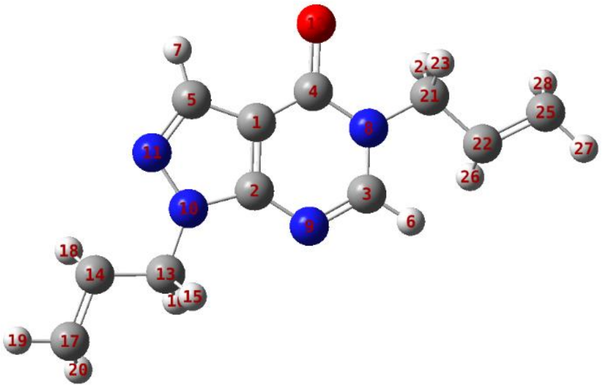

Figure S5: A view of the energy minimized 3D-geometry of PPD specie in the aqueous phase calculated using B3LYP/6-31G(d,p).

Table S1: The potentiodynamic parameters of MS in 1M HCl alone, and with 10^{-3} M PPD, at different temperatures.

T (K)	C_{PPD} (M)	i_{corr} μA/cm^2	θ	IE (%)
**303**±0.5	Blank	**560**±0.013	**-**	**--**
	10^{-3}	32±0.006	0.94	94
**313**±0.5	Blank	**750**±0.9	-	-
	10^{-3}	84±0.17	0.88	88
**323**±0.5	Blank	**2034**±1.256	-	-
	10^{-3}	399±3.544	0.80	80
**333**±0.5	Blank	**2099**±1.578	-	-
	10^{-3}	542±2.955	0.74	74
